# Battery management in IoT hybrid grid system using deep learning algorithms based on crowd sensing and micro climatic data

**DOI:** 10.1038/s41598-025-07868-9

**Published:** 2025-07-07

**Authors:** Srinivasan Rajamani, Arulmozhiyal Ramasamy

**Affiliations:** 1 Department of Electrical and Electronics Engineering, Anna University, Chennai, Tamilnadu India; 2 Department of Electrical and Electronics Engineering, Sona College of Technology, Salem, Tamilnadu India

**Keywords:** Zero export inverter, Super capacitor, Anti-windup proportional integral (AWPI) control, IPWS, Energy storage system, Biomedical engineering, Electrical and electronic engineering

## Abstract

Hybrid Grid System (HGS) installation in small and large residential area has major challenges due to domestic loads. Domestic loads are in different duty cycle such as (i) continuous duty i.e., vehicle charging, (ii) short time duty, (iii) periodic duty and (iv) intermittent duty. In this paper, proposed HGS comprises of Internet of Thing (IOT), Photovoltaic (PV) system and wind system (PWS) with Lithium-Phosphate battery paralleled with Super-capacitor, Deep learning controller with PWS is termed as IOT enabled PWS (IPWS). IPWS has zero export converters, reduces electricity demand on grid. Zero-export inverter avoids excess energy to grid and excess energy stored in super-capacitor. IPWS has crowd sensing for microclimatic conditions data acquisition system. Microclimatic Data is used for tuning zero export converters and Battery Management System (BMS) through IPWS. IPWS controller perform with different hybrid Deep learning algorithm such as (i) SCO-LSTM controller and JO-LSTM based BMS (ii) JO-LSTM controller and HBO-LSTM based BMS (iii) HBO-LSTM controller and SCO-LSTM based BMS. IPWS reduces time and space complexity in controller. Among the proposed methods, IPWS with JO-LSTM/ HBO-LSTM based BMS eliminates output power fluctuations and increases transient stability (TS) and damping ratio (DR). Comparative analysis for DC—link and super-capacitor in IPWS is presented. IPWS with JO-LSTM controller, super-capacitor suits for residence loads and provides 29% improved power factor, reduces harmonics 14%, DR of 6%, and low TS.

## Introduction

A structured electrical network delivers electricity to consumers, which is referred as an electrical grid, power grid, or electrical grid. The conventional power grid has no alterations in its fundamental structure. The demand for energy has increased dramatically in recent decades, necessitates the large-scale, of electricity generation and consumption^1,2^. Increase in electricity use and demand leads to problem such as load-shedding, frequent power outages, and vulnerabilities related to weather and climate change in grid. The International Energy Agency (IEA) predicts energy demand will rise by 30%in year 2040. Investigating and incorporating changes in renewable energy sources in electrical grid can meet the demand^3,4^. These loads needs high transient stability (TS) and damping ratio (DR) which cannot be provided by battery based hybrid grid system. In conventional power grid, electricity is produced and distributed to customers without any awareness of power-consumption of consumers. Lack of knowledge in consumers’ patterns of energy consumption results to wastage of energy^5–7^. As a result, the system operates in an open loop with no user feedback in the form of incentives or warnings, which could change the way of energy usage. Moreover, the system becomes unreliable and ineffective. Furthermore, users’ demand for power leads to compromisation of stability, and safety^8–10^.

IoT based hybrid energy efficient storage system is designed with major components such as solar panel, wind, inverter and Energy Storage System (ESS). ESS consists of BMS^11^. This prototype model is capable to store the energy of 7KWh during peak time. The battery management system monitors and controls the energy storage level in battery^12^. The solar panel generates the power in the range of 5KWh and the output power fed to inverter. This inverter provides AC output to the grid fed by AC isolator^13^. The inverter avoids excess energy generated from the solar panel given to grid and returns the excess energy to energy storage system. The generated electrical energy from multi-blade wind mill (5KVA) is converted as DC and given to inverter. The output of inverter is delivered to grid through AC isolator^14^. Isolators are used for isolating the faulty section. The system consists of harmonic filter and reduces fluctuations and harmonics in the generated power^15^. The changes in electrical parameters are monitored by Power Monitoring System (PMS).The system is operated in both ON grid and OFF grid mode. The entire system parameters are collected and transmitted through Wi-Fi module and data are stored in cloud server. IoT based data are used in controller for controlling the signals of energy storage system.

Three-phase v/f inverter with a power LC filter and an unregulated full bridge rectifier circuit is used in hybrid grid system. The primary single phase AC source circuit topology is used to limit the output voltage’s greater value. An air coil and polypropylene capacitor are used as filter, which performs for a wide frequency range. Due to variable frequency range of the rectified signals as function of command signal, the ferromagnetic coil is avoided. When the inductive load is represented as a serial association of pure coil and resistance, the inner coil resistance is taken into account. The distance between the filter and the load is significant, a power transmission line is included. A simple serial LR impedance model is used for calculation of variations in the system. In order to obtain the output voltage according to a suitable computer command signal, analog input signal over the V/f inverter serves as the input signal of the system.

### Problem statement

Due to high efficiency of modern wind energy conversion systems, nearly all modern wind turbines operates in variable speed mode, necessitates the use of back-to-back power electronic converters to decouple generator dynamics from the grid^16^. Topologies of diode-clamped and capacitor-clamped three-level active rectifiers, as well as feasible switch reductions, are investigated for modern high-power wind energy conversion systems (WECS)^17^. Over the last few decades, model predictive control (MPC) techniques have emerged as a potential power electronics control methodology. Proportional Integral (PI) controller uses discrete-time switching, it is critical to find consistent references for these variables. Thus in this work MPC formulation is proposed for closed-loop control of AFEs. In the process of designing and maintaining a secure power system operation, one of the key limitations is the transient stability^18^. Transient stability refers to the power system’s capacity to sustain synchronism in the face of extreme disruptions. These disruptions may be caused by problems such as a transmission line short circuit, generator failure, load gain or loss, or loss of a section of the transmission network^19^. In case of residential loads such as car charging point has frequent on/off which leads to increase in transient and stability margin^20^.

### Contributions

The aforementioned drawbacks in hybrid grid system have been overcome in the proposed work by using IoT enabled photovoltaic wind system. In this paper, deep learning algorithm is used to monitor the PWM signals sent to the converters and control the operation of the IPWS. This reduces the transient stability due to load variations and controls the damping oscillations. Due to this, the harmonics in the current is reduced which enhances the performances of the battery management system.

(i) To apply optimized deep learning algorithms such as (i) SCO-LSTM (ii) JO-LSTM (iii) HBO-LSTM for tuning the PWM based on crowd-sensing data.
(ii) To apply, algorithms such as (i) SCO-LSTM (ii) JO-LSTM (iii) HBO-LSTM for battery management system for different domestic load conditions and reduce the harmonics
(iii) To reduce, the damping ratio and increase the stability during domestic load, the proposed IPWS based on optimized Deep learning algorithms such as (i) SCO-LSTM (ii) JO-LSTM (iii) HBO-LSTM are applied in PWM controller and BMS.
(iv) To reduce computational complexity of the hardware and for efficient output, same algorithms such as (i) SCO-LSTM (ii) JO-LSTM (iii) HBO-LSTM are applied in PWM controller and BMS.
(v) To evaluate efficiency of the proposed algorithms tested in different domestic loads with traditional algorithms

In this paper, Hybrid deep learning algorithm is used to monitor and control the PWM signals sent to the converters and control the operation of the IPWS based on the converters output voltage, current, crowdsensing and microclimatic data. This control and monitoring of IPWS reduces the transient stability due to load variations and controls the damping oscillations. Due to this, the harmonics in the current is reduced which enhances the performances of the battery management system. Existing systems controls the PWM using PI controllers and never considers the microclimatic data and battery condition parameter data.

## Working principle of proposed system

**Fig. 1:**
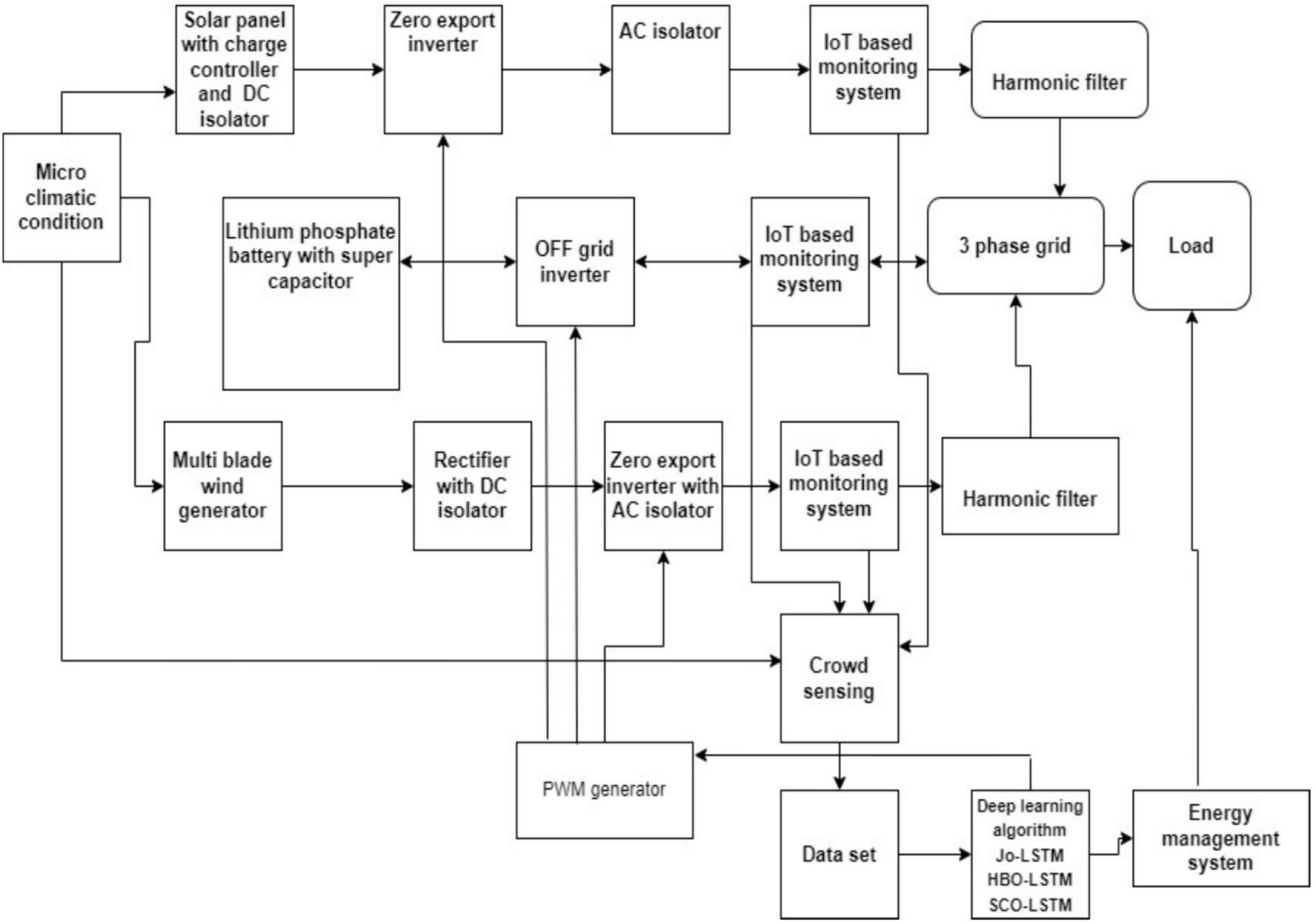
Block diagram of IoT based energy efficient storage system using zero export inverter.

The proposed IPWS hybrid System with IoT based energy efficient storage system using zero export inverter is depicted in Fig. 1.

Modern PV system voltage without batteries is generally from 230 to 610 V. The battery-based PV systems require charge controllers to regulate and stabilize the PV output and ensures the safe of the system. The power generation with high system capacity draws an utmost care of attention about safety. Power producers are increasingly worried about Electrical precautions and protection problems embossed in photovoltaic power plants. Photovoltaic system safety, reliability and stability constant power production provides a profit to power investors and continuity of the power generation. External isolators are provided with the PV systems. Typically, operational voltage of the isolator is equal to or greater than the requirements of the system and selected according to maximum operating power parameters of the PV module. If the PV module is used to size the isolator according to input voltage current curve and ensures the utilization during weather and climatic conditions. Thus, in this work DC isolator is used in IPWS as shown in Fig. 1.

The following equation provides the minimum ratings of the Isolator as in Eq. (1) and (2)

1 $$voltage = ~S_{{p}} *~V_{o} *1.15$$

2 $$Current = P_{p } * I_{S} *1.25$$

where SP: Count of interconnected panels in series, P_p_: Count of interconnected strings in parallel, V_o_-Open-Circuit Voltage, I_s_-Short-Circuit Current.

Hybrid plant needs to be supervised and ensures reliability. In this proposed system, IoT based monitoring system is performed. The Internet of Things (IoT) technology enables mobile device for monitoring and control operation of system. The Internet of Things (IoT) is the interconnection of devices in distant locations.

### Dataset description

In a PV system based on crowd sensing, data is collected from various sources such as IoT-based monitoring systems, smart devices, and user feedback, to enhance energy management and grid stability. The dataset consists of solar panel output parameters such as voltage, current, and power generation efficiency, along with microclimatic data such as solar irradiance, temperature, humidity, wind speed, and atmospheric pressure. Real-time energy consumption data from households and industries is gathered through smart meters, providing insights into demand patterns and device-level energy usage. Additionally, grid interaction data, including energy import/export, frequency variations, voltage stability, and harmonic distortions, is monitored to ensure efficient grid integration. User feedback plays a crucial role in understanding demand-side response patterns, preferred energy usage times, and manual energy-saving inputs. Battery storage data, such as the state of charge (SOC), depth of discharge (DOD), and charge/discharge efficiency of lithium phosphate batteries, is also collected to optimize storage utilization. Furthermore, IoT sensors provide continuous monitoring of smart inverters, AC and DC isolators, and overall system performance, detecting faults and inefficiencies in real time. By leveraging this diverse dataset, deep learning algorithms can predict energy demand, optimize power distribution, and improve the efficiency and reliability of PV-based energy management systems. Table 1 shows Residential load type tested.

**Table 1:** Residential load type tested.

Appliance/device	Typical power consumption (Watts)	Daily usage (Hours)	Daily energy consumption (kWh)
LED light	08-Oct	08-Oct	0.064–0.1
Ceiling FAN	30–70	08-Dec	0.24–0.84
Air Conditioner	1000–2000	04-Aug	Apr-16
Geyser	2000–3000	02-Apr	04-Dec
Refrigerator	100–200	24	2.4–4.8
TV	80–200	04-Jun	0.32–1.2
Computer	65–250	02-Apr	0.13–1
Two-wheeler EV Charging	~ 330 (33 Wh/km for city driving)	~ 3 (for ~ 100 km/day)	~ 1

### Crowd sensing based pwm tuning using deep learning algorithm

Mobile sensing devices are used in crowd sensing, often referred to as participatory sensing or mobile crowd sensing, to gather information about people’s surroundings^21^. Through task division is highly expressive and powerful sensing and used in large-scale sensing. Gaining more participants to gather better quality data is essential to success. In this paper, with the help of crowd sensing device, the PWM signal given to the converter, flow of current and status of the battery management system are monitored^22^. The use of computer-aid tools for protection, monitoring and analyzing the performance of the generation and utilization system is performed using Energy Management System (EMS)^23^. The Energy Management System enhances the adequate use of electricity, commit towards conservation of energy sources and reduces the omission of greenhouse gases^24^. During the peak-load situation, notably this proposed IPWS system secures the available energy and shares among all the devices based on their priority. The constitutes of the energy management system is Charge Controller, Super-capacitor and Battery modules. The prevention of overcharging of the battery system is performed using charge controller. Modern charge controller constitutes a high efficiency operation and maximum power point tracking. The super-capacitor charges and discharges energy rapidly. The super-capacitor has double layers and rated voltage is between from 1 to 3 V.

The Stored capacity of the super-capacitor is in Eq. (3).

3 $${\text{E}} = {\raise0.7ex\hbox{$1$} \!\mathord{\left/ {\vphantom {1 2}}\right.\kern-0pt} \!\lower0.7ex\hbox{$2$}} C_{S } V_{s}^{2}$$

where E is stands for energy captured. C_s_ is the capacitance of the supercapacitor, V_s_ is the supercapacitor rated voltage.

The super-capacitor has more than one million charge cycle of lifespan. The super-capacitor capacitance rating is in the range lies in hundreds of farads. For obtaining the desired capacitance and rated voltage of supercapacitor, it is formed by rows (M_sc_) and columns (N_sc_) module as in Eq. (4) and (5).

4 $${\text{Capacitance}},{\text{ C}} = C_{sc } *\left( {M_{sc} /N_{sc} } \right)$$

and

5 $${\text{Voltage}},{\text{ V}} = V_{SC } * N_{sc}$$

In general, the cost of battery rises as power density range expands. Despite having the same power density, super-capacitors are less expensive than standard capacitors. A Li-Polymer battery has a much lower energy density than Li-ion battery.

## Optimized deep learning algorithm

The complexity of real-world optimization problems has increased due to the recent 10 years’ tremendous advancements in science and technology, which has prompted the development of quick and effective optimization algorithms. Finding the best outcome possible in a given situation is known as optimization. Optimizing the intended outcome or reduces the amount of work is done through optimization algorithm. Optimization is the process of identifying the appropriate variables that provide the minimal or maximum value of an objective function f(x), given that the desired benefit or the required effort has been stated as an objective function. Because optimization techniques are important, lot of novel meta-heuristic optimization algorithms have been developed in the last few decades. In this work, three different optimization algorithms has been discussed and analyzed such as Human behavior-based optimization (HBO-LSTM), jellyfish optimization (JO-LSTM) and single candidate optimization (SCO-LSTM) for PWM generation based on crowd sensing, microclimatic data and battery management system.

### Human behavior-based optimization (HBO)

A person accomplishes all their goals is considered as successful. A person needs to strive to be the best version of himself, if he wants to achieve goals. Everyone has a unique perspective, everyone approaches success differently and chooses to accomplish certain goals in order to succeed. Every member of a society has interests outside of their line of work. For instance, in addition to their primary interest, electrical engineers might also like painting. Many people in our society hold a variety of opinions, yet these opinions change during the course of a person’s life. Everyone makes a range of relationships during his life and uses those contacts’ thoughts and counsel to better his own life. These interactions has been viewed as meetings with advisors, which may or may not be fruitful. HBO algorithm generates the initial individuals, they are dispersed among several fields. Every human aspires to become better person in every profession through education, and then they locate a representative of society at large and begin to confer with him. Furthermore, people’s beliefs might change, leads to change careers or areas of study. In this algorithm, field’s changing likelihood is considered, an individual in some professions may find more appropriate path and change fields. In the end, the algorithm will verify the stopping conditions and stop if any of them are reached^25^. The complexity analysis includes initialization, education step, consultation step, field changing probability and iterative nature. HBO has unique features, such as consultation and field-changing mechanisms, make more adaptive potentially more computationally expensive in scenarios with large populations or high-dimensional problems.

### Jellyfish optimization (JO)

Across the world, jellyfish is found in water with a range of temperatures and depths. They have bell-like shape, with some having a diameter of less than a centimeter and others having a very big one. They come in an extensive range of colors, dimensions, and forms. Every one of the numerous species shows unique adaptations to the marine environment. Swarm formation is influenced by a variety of elements, such as temperature, predation, oxygen availability, accessible nutrients, and ocean currents. These phenomena have allowed the jellyfish species to spread out practically everywhere in the ocean, in addition to the individual movements of each jellyfish inside the swarm and their subsequent migrations along the ocean current produces jellyfish blooms^26^. The optimal location is found by comparing food quantities because jellyfish visit different areas with varying amounts of food. Thus, new method is proposed that draws inspiration from the search behavior and oceanic movements of jellyfish. The jellyfish optimization (JO) algorithm comprises of three steps, they are:

(i) A “time control mechanism” controls when the jellyfish switch between moving inside the swarm or following the ocean current.
(ii) In the ocean, jellyfish migrate in search of food. They are drawn to areas with larger food supply availability.
(iii) The location and related objective function govern the amount of food found.

The pseudo code for JO-LSTM is as follows:

**Figure:**
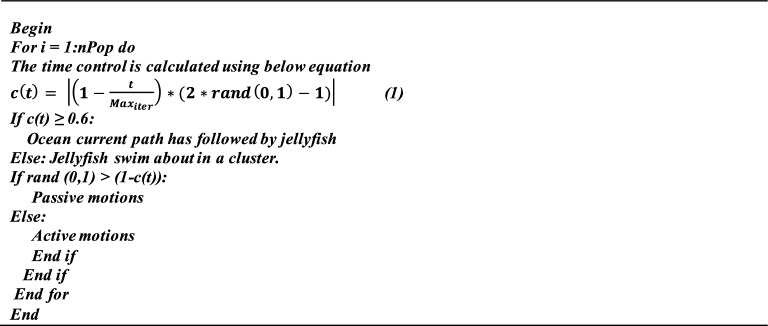
Algorithm of Jellyfish Optimization.

The complexity analysis of Jellyfish Optimization (JO) includes initialization, objective function evaluation, exploration and exploitation, boundary checking and convergence. Jellyfish Optimization algorithm and its variants typically exhibit a time complexity makes them computationally efficient for many real-world applications, and maintaining simplicity in implementation.

### Single candidate optimization (SCO)

The suggested scheme divides the whole optimization process, which consists of 'T' function evaluations or iterations, into two phases. In each step, the candidate solution updates its position in a different way. The two well-known meta-heuristic optimization techniques that have been applied independently are single-solution-based algorithms and two-phase approaches. The single candidate technique and the two-phase strategy are combined in the established method and creates a single, reliable algorithm. Primarily, the suggested algorithm uses a distinct collection of formulas to adjust the candidate solution’s location based on data, that is, its present location. The two-phase approach aims to balance exploration and exploitation while offering diversity^27^.

**Figure:**
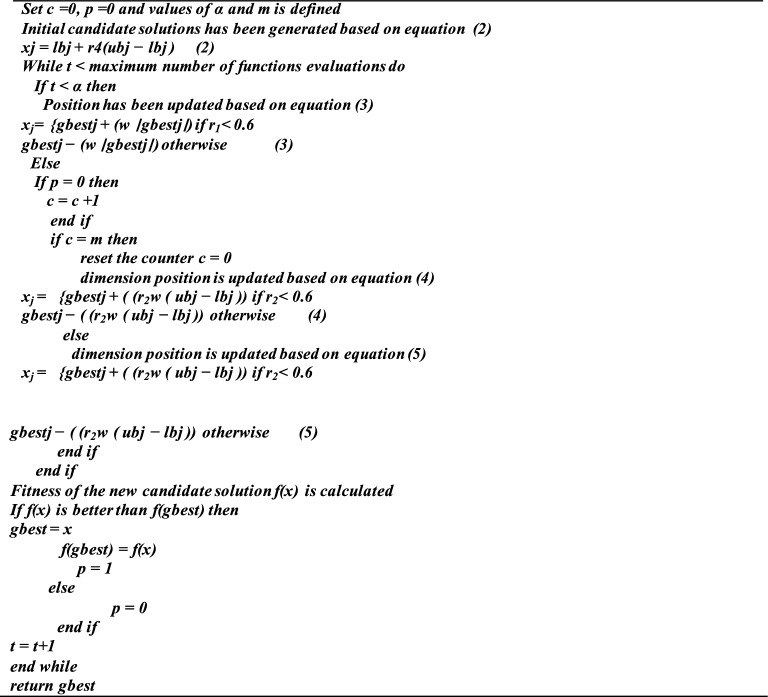
Algorithm of SCO.

The complexity analysis of Single Candidate Optimization (SCO) include time complexity and space complexity. SCO is computationally efficient with low time and space complexity compared to population-based methods. However, performance can vary based on problem characteristics and implementation details. Training Process of Jellyfish Optimization (JO): Inspired by the movement and foraging behavior of jellyfish, JFO explores and exploits the search space efficiently. The algorithm follows three primary phases: passive motion, active motion, and swarm dynamics. Training process of Single Candidate Optimization (SCO): A minimalistic approach that optimizes using a single solution and iteratively refines it using perturbation techniques. Training process of Human Behavior-Based Optimization (HBO): Models human decision-making processes such as social influence, exploration, and experience-driven adaptation.

## Performance analysis of zero inverter capacitor with SCO-LSTM controllers

**Fig. 2:**
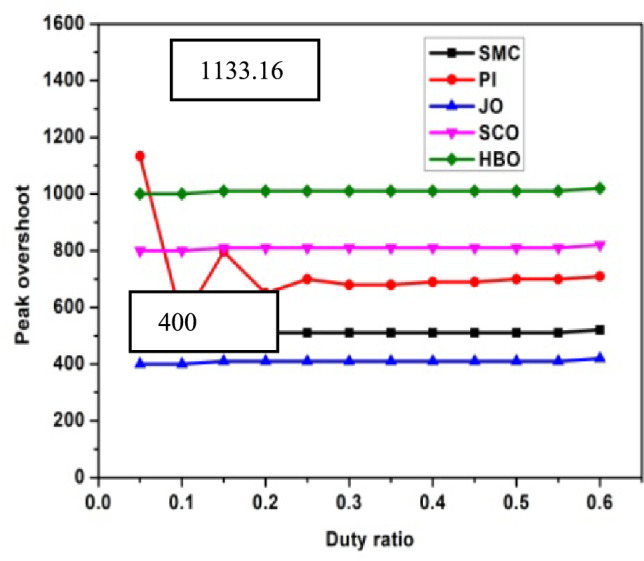
Peak Overshoot for SCO-LSTM and HBO-LSTM Controller with DC link capacitor.

**Fig. 3:**
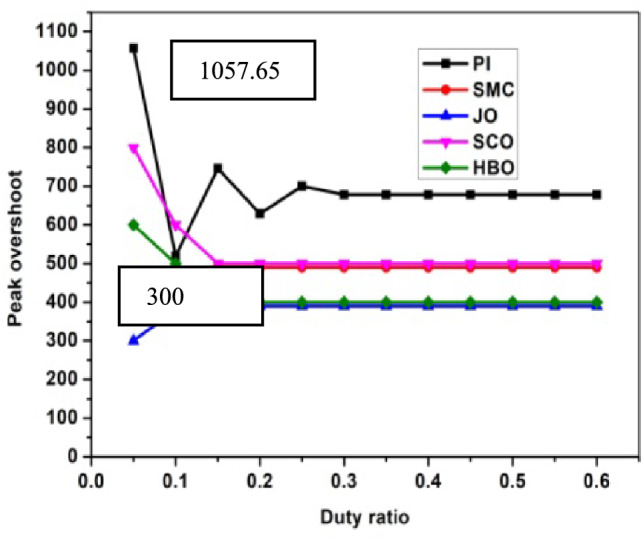
Peak Overshoot for JO-LSTM and HBO-LSTM Controller with SC.

**Fig. 4:**
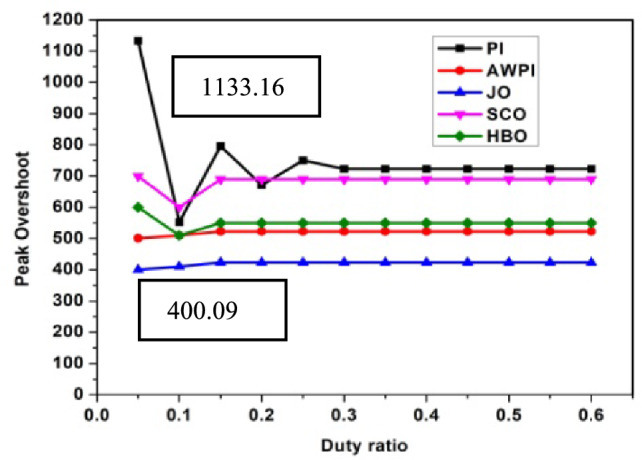
Peak Overshoot for SCO-LSTM and HBO-LSTM Controller with DC link capacitor.

**Fig. 5:**
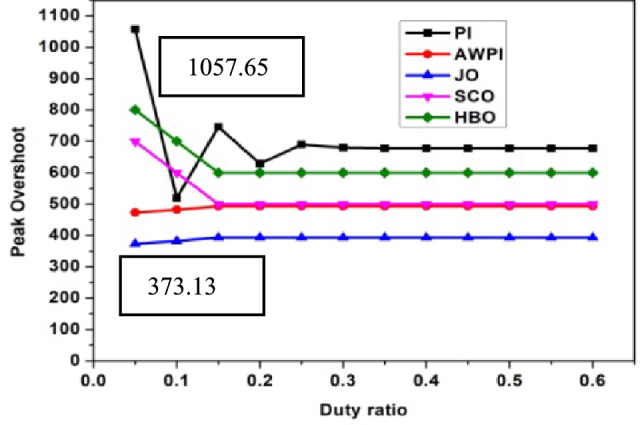
Peak Overshoot for SCO-LSTM and HBO-LSTM Controller with SC.

**Fig. 6:**
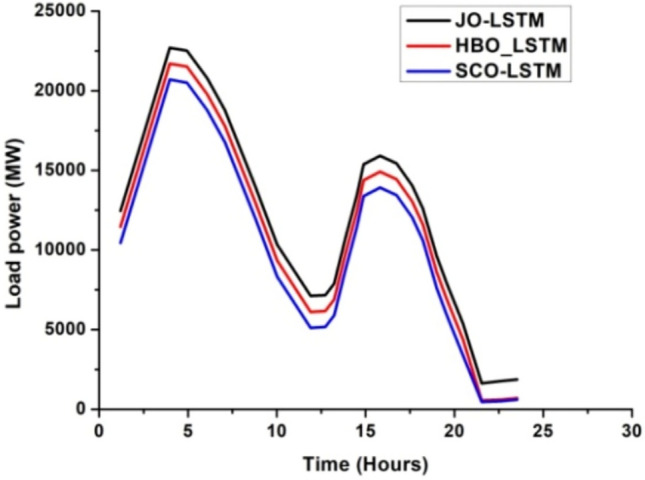
Load power (MW) Vs time (hours).

The performance analysis of three controllers with DC link capacitor and Ultra-capacitor are analyzed and shown in Figs. 2, 3, 4, 5 and 6. The peak overshoot variations for different duty ratio for DC-link capacitor with PI and Sliding Mode Controller (SMC) controller are shown in Fig. 3. The peak overshoot variations for different duty ratio for super capacitor with PI and SMC controller is shown in Fig. 4.

Figure 2 depicts the analysis of SCO-LSTM controllers with DC link capacitors. From Fig. 2, it is implied that the peak overshoot values of DC-link with PI controller is 1133.16 and with JO-LSTM controller is 400. From Fig. 3, it is observed that the peak overshoot for the identical two controllers with super-capacitors is 1057.65 and 300. Peak overshoot is reduced as a result of the controller change, and it is reduced further by using an ultra-capacitor instead of a DC link capacitor. The PI controller has a higher peak overshoot than the other controllers. The peak overshoot variations for different duty ratio for DC-link capacitor with different controllers is shown in Fig. 4. The peak overshoot variations for different duty ratio for super capacitor DL controller are shown in Fig. 5.

Figure 4 shows the maximum peak overshoot values of DC-link capacitor with PI controllers is 1133.16 and JO controllers is about 400.09. As shown in Fig. 5, the peak overshoot of the same two controllers with supercapacitors is 1057.65 and 373.13.Peak overshoot is reduced as a result of the controller change, and reduced further by using an super-capacitor instead of a DC link capacitor. The PI controller has a large peak overshoot, when compared to other controllers. From Figs. 2, 3, 4 and 5, it is concluded that the proposed system works well with JO-LSTM controller linked with super capacitor. The performance of SCO-LSTM, JO-LSTM and HBO-LSTM for PWM tuning is listed in Table 2.

**Table 2:** Performance of SCO-LSTM, JO-LSTM and HBO-LSTM for PWM tuning.

Sno	SCO-LSTM	JO-LSTM	HBO-LSTM	Random forest^28^	Bayesian classifier^29^	Fuzzy SVM^30^
Specificity	**0.8**	**0.9**	**0.85**	0.7	0.7	0.8
Sensitivity	**0.8**	**0.9**	**0.85**	0.75	0.85	0.7
Precision	**0.7**	**0.9**	**0.75**	0.8	0.85	0.7
Recall	**0.7**	**0.85**	**0.75**	0.85	0.8	0.75
Accuracy	0.7	0.9	0.8	0.75	0.8	0.85

Significant values are in (bold).

### Performance analysis of BMS using JO-LSTM

**Fig. 7:**
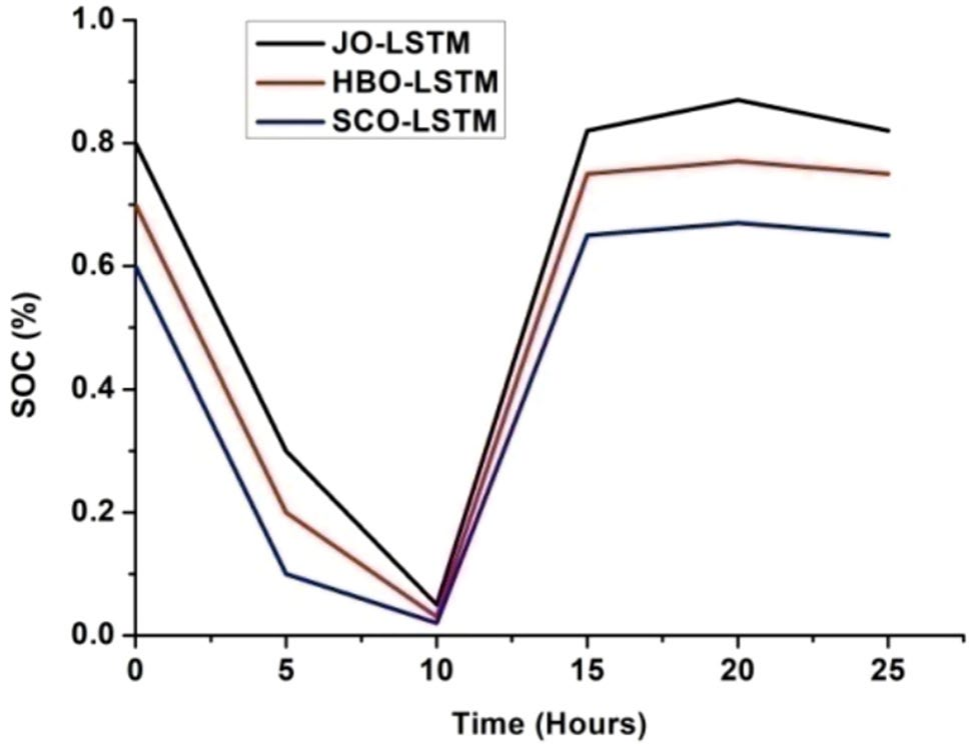
SoC (%) Vs time (hours).

**Fig. 8:**
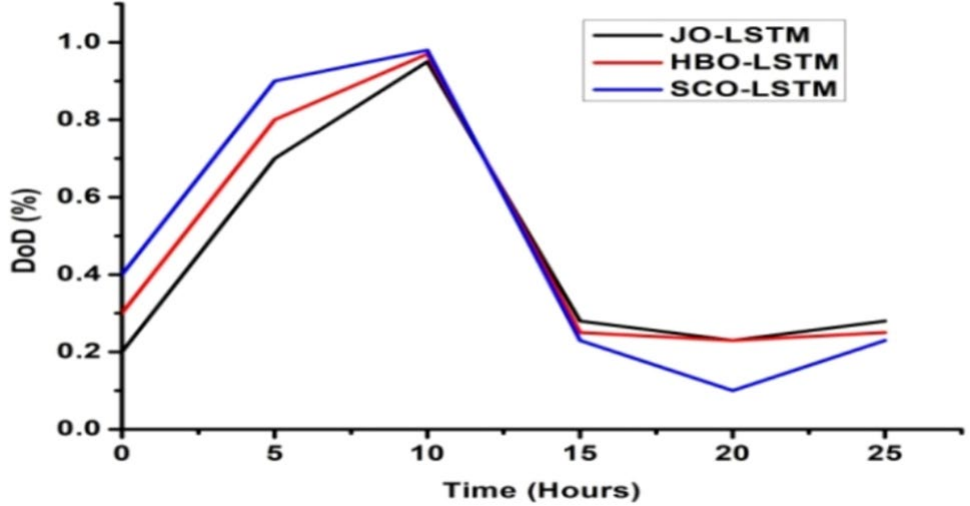
DoD (%) Vs time (hours).

The deep learning algorithm has been used to monitor and stabilize the battery and system load power which in turn enhances the system performances^31–33^. The above discussed LSTM algorithm has been implied to the proposed hybrid system and the results are plotted. The graphical representation of load power versus time, battery state of charge (SoC) versus time and battery depth of discharge (DoD) versus time is depicted in Figs. 6, 7 and 8.

From Figs. 6, 7 and 8, it has been observed that with JO-LSTM algorithm, the system performance has been improved and provides the stable and uninterrupted power supply to the load^34–38^. The battery characteristics has been maintained properly with this proposed model. The comparative analysis of all three LSTM models has been listed in Table 3

**Table 3:** Comparative analysis of deep learning algorithms.

Parameters	LSTM model		
	HBO	JO	SCO
Load power (MW)	21,000	22,000	20,000
SoC (%)	0.7	0.8	0.6
DoD (%)	0.3	0.2	0.4

From Table 3, it has been inferred that the JO-LSTM model achieves high peak load power and maintains the battery characteristics. Thus, the jelly fish model is preferred compared with other two algorithms.

## Result analysis of JO-LSTM controller and SCO-LSTM based BMS

In this proposed DL controller system, the DC link capacitor is restructured with a ultra-capacitor, which maintains the DC voltage at the DC link for improved system outcomes such as settling time and DC link voltage^39–43^.

**Fig. 9:**
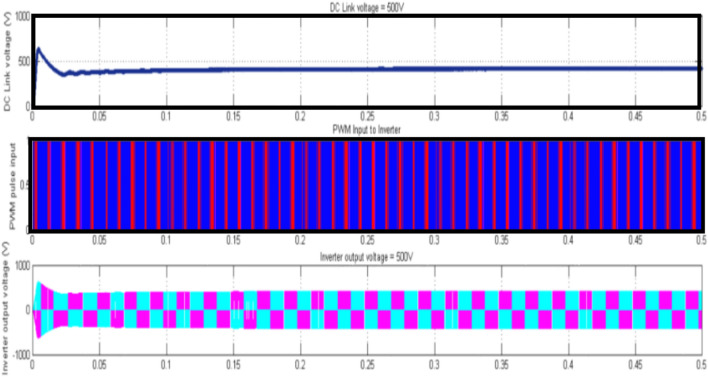
Mode 3Solar and SCO-LSTM controller with HBO-LSTM based BMS.

Figure 9 depicts a solar system with a SC link capacitor that contributes a DC link voltage of 500 V, an inverter yield of 500 V, and a settling time of 0.5 s, in three-phase power.

**Fig. 10:**
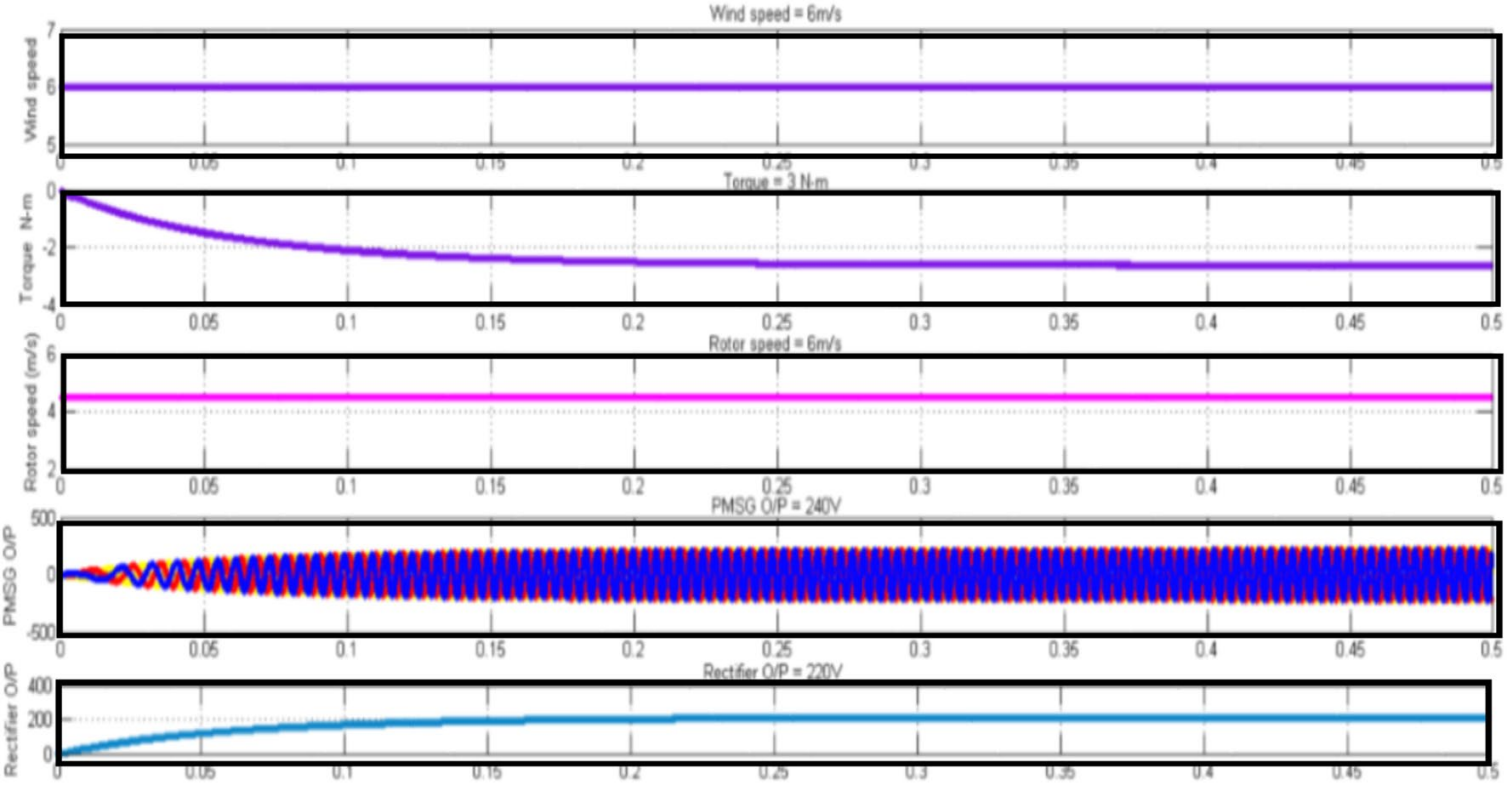
Mode 3Wind and HBO-LSTM with HBO-LSTM based BMS.

Figure 10 depicts the wind system with super capacitor that contributes a DC link voltage of 240 V and an inverter yield of 220 V with a settling time of 0.5 s in three phase power.

**Fig. 11:**
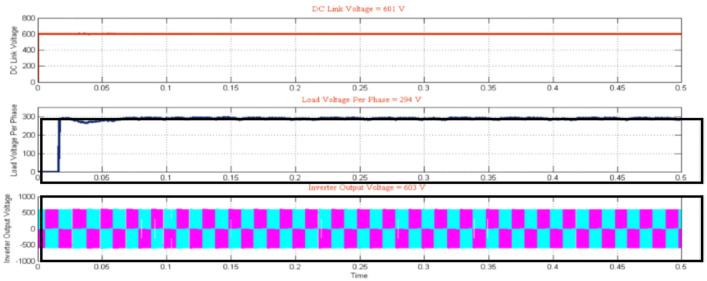
Mode3 Hybrid with Load–AWPI Controller with JO-LSTM with HBO-LSTM based BMS.

As shown in Fig. 11, the hybrid system contributes a DC link voltage of 601 V, an inverter yield of 500 V with a settling time of 0.5 s, and load voltage per phase of 294V. The detailed specifications of the solar, wind, hybrid system and inverter system are shown in Table 4 with the load voltage per phase accompanied the system with Supercapacitor.

**Table 4:** Detailed specification of the hybrid system with load voltage.

Wind specifications	Solar specifications
Wind speed–6 m/Sec	DC Link Voltage—500 V
Torque—3N-M	Inverter Output Voltage—500 V
RotorSpeed—6 m/S	HYBRIDSPECIFICATIONS
PMSG output—240 V	DC Link Voltage—601 V
Rectifier output—220 V	Inverter Output Voltage—603 V
load voltage per phase with Super-capacitor—294V	

**Fig. 12:**
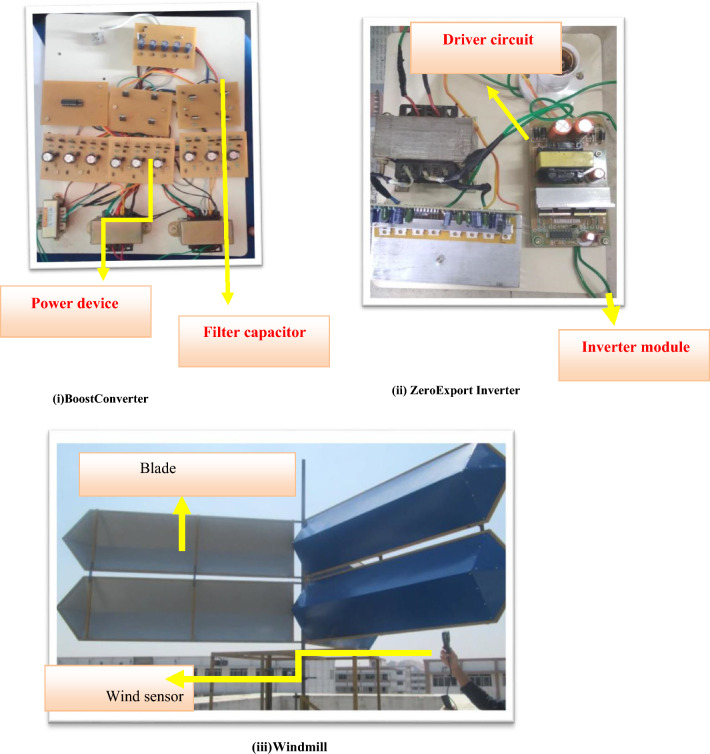
(i) BoostConverter. (ii) ZeroExport Inverter. (iii)Windmill.

**Fig. 13:**
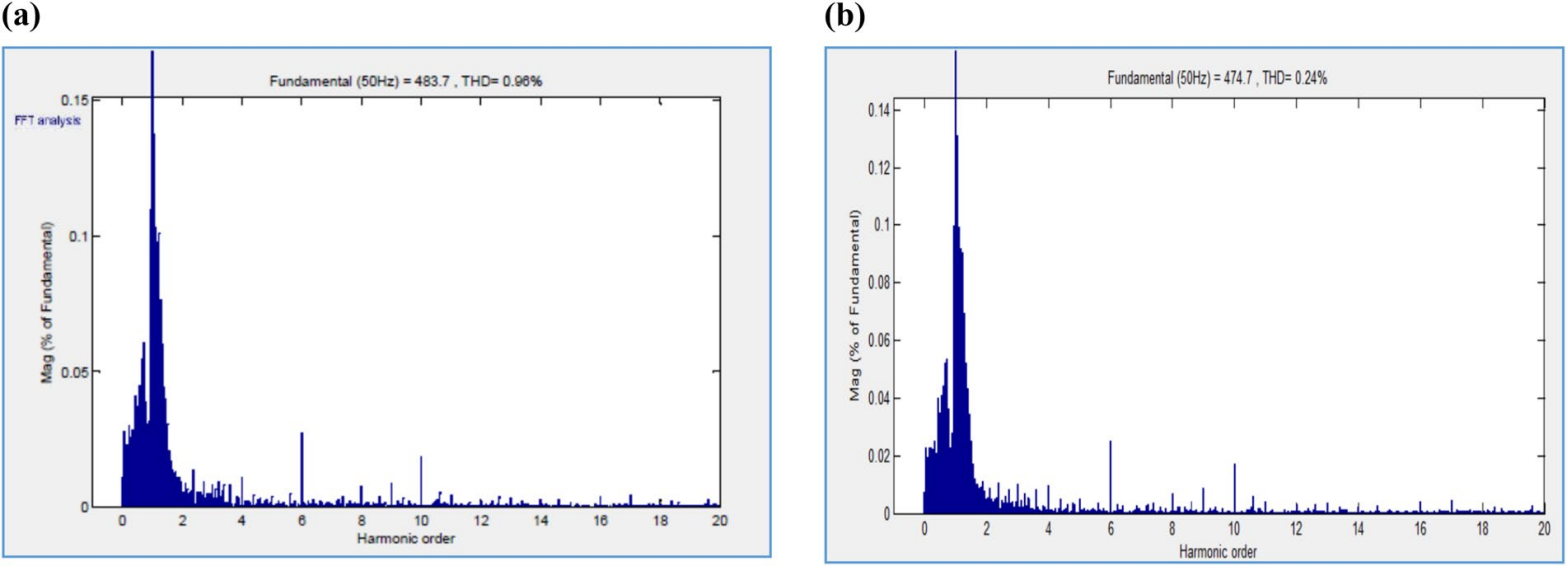
(**a**) THD values of Conventional Inverter with Supercapacitor. (**b**) THD values of ZE Inverter with Supercapacitor.

**Fig. 14:**
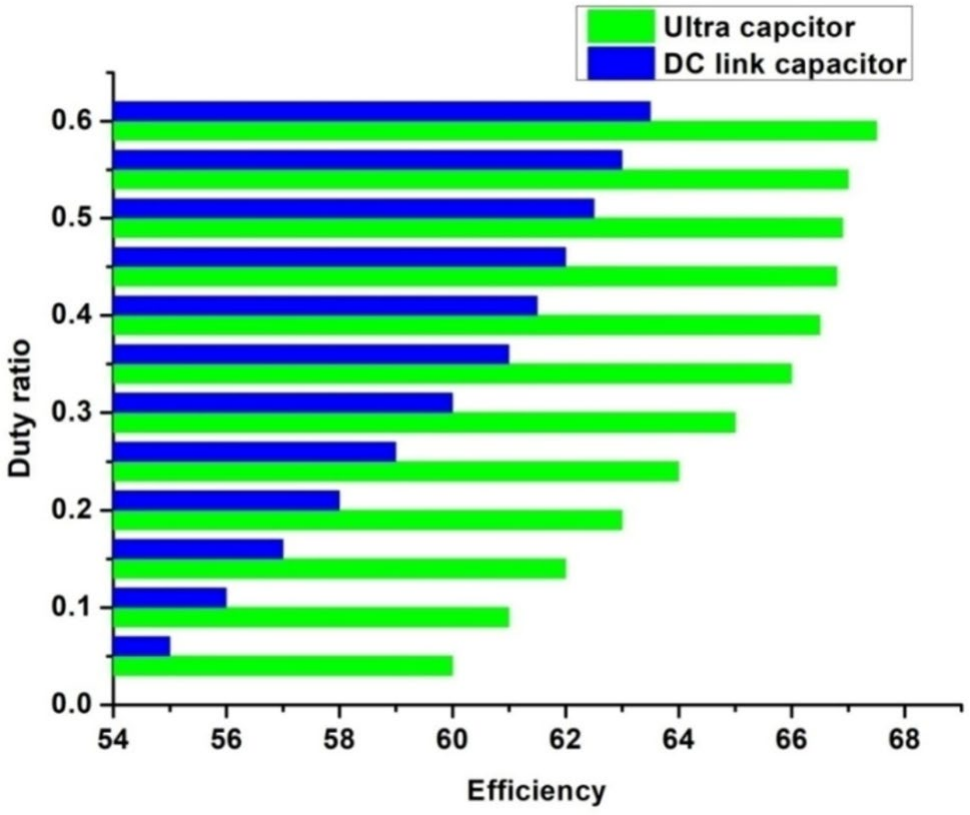
Analysis of efficiency with DC link capacitor and super capacitor.

A prototype model of boost converter, zero export inverter and wind mill is depicted in Fig. 12. Figure 13a depicts the THD value of 0.95 for conventional inverter, whereas Fig. 13b depicts the THD value of 0.24 for the proposed system with zero export inverter and harmonic filter. The pictorial representation of efficiency analysis between super capacitor and DC-link capacitor is depicted in Fig. 14.

From Fig. 14, it is implied that the AWPI controller has improved efficiency with super-capacitor compared to DC link capacitor. The performance of the proposed optimization algorithms is evaluated as in Table 5. The overall comparison of for DC-link capacitor and super capacitor is listed in Table 6.

**Table 5:** Performance Evaluation Metrics.

Metric	Jellyfish optimization	Single Candidate optimization	Human behavior-based optimization
Convergence speed	Fast due to swarm intelligence	Moderate, depends on initial solution	Fast due to adaptive learning
Accuracy	High, as it explores and exploits well	Moderate, prone to local optima	High, learns from past experiences
Computational complexity	Moderate (O(N log N))	Low (O(N))	Moderate to High (O(N^2))
Robustness	High, adapts to dynamic environments	Low, sensitive to initialization	High, accounts for social interactions
Stability	Consistent across multiple runs	Variable, depends on tuning	Stable due to behavioral modeling
Scalability	Suitable for high-dimensional problems	Works best for low-dimensional problems	Scales well with increasing complexity

**Table 6:** Comparative analysis of DC-link capacitor and super capacitor.

	DC-LINK CAPACITOR	SUPER CAPACITOR
Efficiency (%)	63	67

From Table 6, it is implied that the efficiency of the system is enhanced with super-capacitor compared with DC-link capacitor. Table 7 shows Performance of SCO-LSTM/JO-LSTM/HBO-LSTM based controller and BMS.

**Table 7:** Performance of SCO-LSTM/JO-LSTM/HBO-LSTM based controller and BMS.

Statical parameters	SCO-LSTM controller & JO-LSTM based BMS	JO-LSTM controller & HBO_LSTM based BMS	HBO-LSTM controller & SCO-LSTM based BMS
Specificity	0.8	0.9	0.85
Sensitivity	0.8	0.9	0.85
Precision	0.7	0.9	0.75
Recall	0.7	0.85	0.75

From Table 7, it is implied that with JO-LSTM controller and HBO-LSTM based BMS enhances the performance of the BMS.

## Experimental validation

**Fig. 15:**
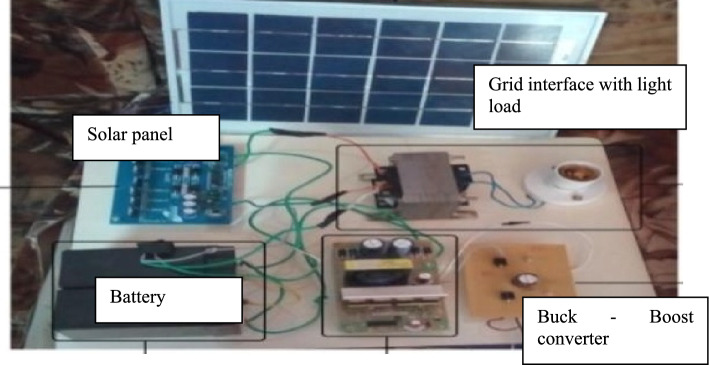
Hardware Model.

The Fig. 15 displays the hardware model of hybrid grid connected zero export inverter system with supercapacitor. In this model, the fluctuating voltage from the solar module and wind mill is given to grid through boost converters and zero export inverters.

The peak value of DC link capacitor is 1098.23. The DC link capacitor is replaced with super capacitor value of 3F in order to reduce the peak overshoot. The peak value for the Super capacitor is 1022.91. Thus, with super capacitor, the peak overshot value is greatly reduced. The simulated and empirical values are listed in Table 8.

**Table 8:** Contrast Index of DC link voltages and Supercapacitor Voltages.

From replica results						From emperical results					
S No	Kind of controller	Voltage with DC link capacitor		Voltage with super capacitor		S.No	Kind of controller	Voltage With DC link-capacitor		Voltage with super-capacitor	
1	JO	Peak overshootvalue	InverterDC Input value	Peak overshoot value	InverterDC Input value	2	AWPI	Peak overshoot value	Inverter DC Input value	Peak overshoot value	Inverter DC Input value
		**1134.18 V**	**718.62 V**	**1057.61 V**	**677.12 V**			**1098.23 V**	**699.25 V**	**1022.91 V**	**666.73 V**

Significant values are in (bold).

**Fig. 16:**
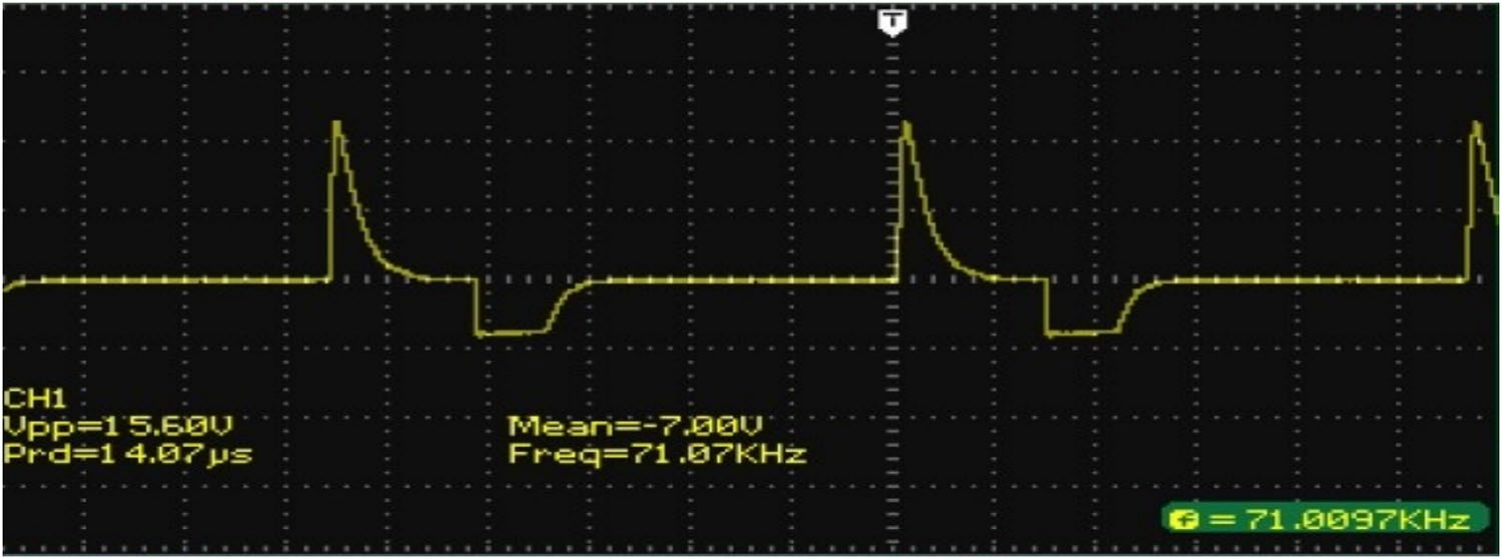
Supercapacitor link voltage.

The output performance and results are obtained from the Real time system and monitored in Digital Signal Oscilloscope (DSO) as well as monitored by IoT. IPWS improves the DC link voltage and rectify the changes in DC voltage that were previously processed by the system’s inverter section. The output voltage of the Super capacitor system is 666.73 and it is depicted in Fig. 16.

Table 9 shows the Comparative analysis of DL algorithms.

**Table 9:** Comparative analysis of DL algorithms.

DL algorithms	Residential load capacity (Watt)	THD (%)	Power Factor (PF)	Damping ratio	Transient stability
Simulation analysis of the DL algorithms					
SCO-LSTM controller & JO-LSTM based BMS	500	25	0.7	0.25	HIGH
JO-LSTM controller & HBO-LSTM based BMS	500	11	0.99	0.19	LOW
HBO-LSTM controller & SCO-LSTM based BMS	500	15	0.8	0.15	MEDIUM
Test bed results for the proposed algorithms					
SCO-LSTM controller & JO-LSTM based BMS	500	23	0.6	0.24	HIGH
JO-LSTM controller & HBO-LSTM based BMS	500	11	0.97	0.17	LOW
HBO-LSTM controller & SCO-LSTM based BMS	500	14	0.7	0.15	MEDIUM

From Table 9, it is concluded that the proposed IPWS with JO-LSTM controller and HBO-LSTM based BMS has reduced THD, improved power factor and damping ratio. Thus, the proposed system is well suits for residences.

The Table 10 provides performance metrics such as response time and reliability, along with real-world testing scenarios for battery management systems using the proposed JO-LSTM controller for PWM and HBO-LSTM for BMS.

**Table 10:** Performance metrics of battery management systems for JO-LSTM controller for PWM and HBO-LSTM for BMS.

Parameter	Response time	Reliability (%)	Description
Fast charging	< 50 ms	99.9	Evaluates how quickly the system adapts to fast charging scenarios while maintaining battery health
Temperature variation	< 100 ms	99.5	Assesses the system’s ability to adjust power management under varying temperature conditions
Load balancing	< 70 ms	99.7	Measures how efficiently the system redistributes energy between battery cells to avoid overloading
Fault detection	< 80 ms	99.8	Tests the system’s capability to identify and mitigate battery faults in real-time
Energy efficiency	< 60 ms	99.9	Analyzes power consumption optimization in various operational conditions

The Table 11 provides performance metrics of proposed JO-LSTM controller for PWM and HBO-LSTM for BMS.

**Table 11:** Performance metrics of proposed JO-LSTM controller for PWM and HBO-LSTM for BMS.

Parameter	statistical test	Test statistic (value)	*p*-value	Interpretation
Energy generation (PV & Wind)	T-test/ANOVA	F = 5.62	0.014	Significant variation in energy output under different conditions (*p* < 0.05)
Environmental data impact	Pearson Correlation	r = 0.85	0.001	Strong correlation between solar irradiance and energy output (*p* < 0.05)
Grid stability (voltage/frequency variations)	F-test (Levene’s Test)	F = 2.15	0.087	No significant variance in grid stability (*p* > 0.05)
Battery performance (SOC, DOD, efficiency)	Paired T-test	t = 3.98	0.008	Significant improvement in battery performance after optimization (*p* < 0.05)
IoT-based monitoring accuracy	Wilcoxon Signed-Rank Test	W = 175	0.022	IoT-based monitoring accuracy improved significantly (*p* < 0.05)
Harmonic distortions in grid	Kruskal–Wallis Test	H = 10.21	0.018	Significant reduction in harmonic distortions post-filtering (*p* < 0.05)
Deep learning algorithm performance (JO-LSTM, HBO-LSTM, SCO-LSTM)	ANOVA (Post-hoc Tukey Test)	F = 7.89	0.004	Significant difference in algorithm accuracy, favoring SCO-LSTM (*p* < 0.05)
Crowd-sensing data reliability	Cohen’s Kappa/Fleiss’ Kappa	κ = 0.78	–	High agreement among crowd-sensing sources (κ > 0.75 is considered strong)
Overall system efficiency	Regression Analysis	R^2^ = 0.92	< 0.001	Strong predictive power of key parameters affecting system optimization

The following Table 12 provides the computational complexity of key deep learning algorithms along with references and citations for further reading.

**Table 12:** Deep learning algorithm complexity analysis.

Algorithm	Training complexity	Inference complexity	Reference
Multilayer perceptron (MLP)	O(N * D * H + H^2)	O(H * D)	^44^
Convolutional neural network (CNN)	O(N * (D * K^2 * C))	O(D * K^2 * C)	^45^
Recurrent neural network (RNN)	O(N * T * H^2)	O(T * H^2)	^46^
Transformer	O(N * d^2 + N^2 * d)	O(N * d^2)	^47^
Graph neural network (GNN)	O(N * E * H^2)	O(E * H^2)	^48^
JO-LSTM	O(N * T * H^2)	O(T * H^2)	Proposed

Explanation of Notation: N represents Number of training samples, D represents Input data dimension, H represents Number of hidden units, K represents Kernel size (for CNNs), C represents Number of channels (for CNNs), T represents Time steps (for RNNs and LSTMs), d represents Feature dimensionality (for Transformers) and E represents Number of edges in the graph (for GNNs)^49–52^.

## Conclusion

In this work, the performance of the IoT enabled photovoltaic and wind system has been observed and analyzed. The lithium-Phosphate battery has been used as a backup in parallel with supercapacitor. The simulation analysis has been carried out between DC-link and super capacitor and found that with super capacitor, the energy efficiency has been enhanced. The deep learning controller has been used for PWM tuning and BMS. Hybrid grid system (HGS) enhances the energy efficiency in residences through proposed IPWS system, which comprises of Battery management systems (BMS) and PWM tuning. BMS and PWM tuning are performed with proposed Deep learning algorithm such as (i) SCO-LSTM controller & JO-LSTM based BMS (ii)JO-LSTM controller &HBO-LSTM based BMS (iii) HBO-LSTM controller & SCO-LSTM based BMS reduces time and space complexity in the controller. From the analysis, it has been inferred that the JO-LSTM controller have low THD, minimizes the peak overshoot value, the settling time to achieve the final value, and the system’s stability. IPWS eliminates output power fluctuations and increases TS and DR. IPWS with JO-LSTM controller, super-capacitor suits for residence loads and provides 29% improved power factor, reduces harmonics 14%, DR of 6%, low TS, when compared with traditional methods. Thus, the proposed IPWS maximizes the output energy and reduces the output power fluctuations. A prototype model has been built to validate the simulation results. Hence, the proposed hybrid system suits for residences. Furthermore, partial shading can be included with proposed deep learning controller.

## Data Availability

'The datasets used and/or analysed during the current study available from the corresponding author on reasonable request.'
